# Psychophysiological Responses to Competition and the Big Five Personality Traits

**DOI:** 10.2478/v10078-012-0057-x

**Published:** 2012-07-04

**Authors:** Erdal Binboga, Senol Guven, Fatih Çatıkkaş, Onur Bayazıt, Serdar Tok

**Affiliations:** 1 Ege University, Faculty of Medicine, Department of Biophysics, Izmir, Turkey.; 2 Kirazdagı Primary School, Derepazarı, Rize, Turkey.; 3 Bartin University, School of Physical Education and Sports, Bartin, Turkey.; 4 Izmir University, Faculty of Medicine, Medical Park Hospital, Department of Biophysics, Izmir, Turkey.; 5 Ege University, School of Physical Education and Sport, Izmir, Turkey.

**Keywords:** Personality, electrodermal activity, sport psychology

## Abstract

This study examines the relationship between psychophysiological arousal, cognitive anxiety, and personality traits in young taekwondo athletes. A total of 20 male and 10 female taekwondo athletes (mean age = 18.6 years; ± 1.8) volunteered for the study. The Five Factor Personality Inventory and the state scale of the Spielberger State-Trait Anxiety Inventory (STAI) were used to measure personality and cognitive state anxiety. Electrodermal activity (EDA) was measured twice, one day and approximately one hour prior to the competition, to determine psychophysiological arousal. Descriptive statistics, Pearson product-moment correlations, and stepwise regression were used to analyze the data. Several “Big Five” facets were related to the EDA delta scores that were measured both one day and one hour before the competition. Two stepwise regressions were conducted to examine whether personality traits could significantly predict both EDA delta scores. The final model, containing only neuroticism from the Big Five factors, can significantly explain the variations in the EDA delta scores measured one day before the competition. Agreeableness can significantly explain variations in the EDA delta scores measured one hour before the competition. No relationship was found between cognitive anxiety and the EDA delta scores measured one hour before the competition. In conclusion, personality traits, especially agreeableness and neuroticism, might be useful in understanding arousal responses to competition.

## Introduction

Hardy’s (1990) catastrophe model is one of the most influential approaches for examining the relationship between competitive anxiety and athletic performance. The model aims to explain how athletic performance can be affected by the interaction between cognitive anxiety and physiological arousal. Hardy et al. (2007) proposed several interactions between cognitive anxiety, physiological arousal, and athletic performance: (a) Cognitive anxiety has a positive relationship with performance when physiological arousal is low; (b) cognitive anxiety has a negative relationship with performance when physiological arousal is high; (c) when cognitive anxiety is low, physiological arousal has an inverted U-shaped relationship with performance; and (d) when cognitive anxiety is high, increased levels of physiological arousal lead to a catastrophic drop in performance (Hardy et al., 2007).

Several studies have tested Hardy’s theoretical framework and provided some empirical support for the predictions of the catastrophe model (Hardy, 1990; Hardy et al., 1994; 2007). However, studies based on the catastrophe model have been criticized as well. The most important point in these criticisms was the method used to manipulate physiological arousal. In these studies, subjects’ physiological arousal was manipulated by shuttle runs that continued until participants’ heart rates increased to a predetermined level, which may not be the same as manipulation by psychological stress (Hardy, 1999; Hardy et al., 2007). The determination of physiological arousal in the studies examining the catastrophe model might be another concern that can limit the model’s ability to predict athletic performance. Only heart rate was measured as an index to control physiological arousal. However, more comprehensive psychophysiological methods may allow for a better understanding of the catastrophe model’s prediction about the relationship between cognitive anxiety, physiological arousal, and athletic performance. For example, taking autonomic reactions into account, which are an important component of emotional response (Mardaga et al., 2006), may make it easier to understand the effect of physiological arousal on performance. These autonomic reactions associated with emotional responses can be described as involuntary, unconscious functions of internal systems and organs (cardiovascular, respiratory, urinary, reproductive, and gastrointestinal systems; liver, smooth muscles, salivatory, sweat, and some endocrine glands) which are mediated by the peripheral part of the nervous system. Many stressors or affective changes in humans evoke these autonomic reactions (Goldstein, 2001). The most commonly used method to evaluate these autonomic reactions is based on electrodermal (sweat gland) responses (Mauss and Robinson, 2009). Lang et al. (1998) also suggested that there is consistency with neural imaging results and electrodermal skin responses to pleasant and unpleasant images. They also suggested that electrodermal skin responses are highly correlated with arousal ratings in subjects (Lang et al., 1998). Variations in electrodermal activity (EDA) depend on the quantity of sweat secreted by the eccrine sweat glands. These glands are mainly located in the hypodermis of the palmar and plantar regions, and they generate sweat excreted through ducts (Groscruth, 2002). In this respect, EDA, which is a classical and sensitive way to assess affective arousal (Dawson et al., 2000), might be a possible physiological parameter for further understanding the relationship between anxiety-induced arousal and performance in well-controlled experimental studies.

In addition to concerns regarding the determination of physiological arousal, the lack of information about the relationship between physiological arousal induced by competition and cognitive anxiety might be another concern in studies based on the catastrophe model. Furthermore, only the relationship between physiological arousal and cognitive anxiety was considered (Hardy, 1999; Hardy et al., 2007). However, in these studies, personality traits that can be related to physiological arousal or that mediated the relationship between cognitive anxiety and physiological arousal were ignored.

In this respect, there is substantial evidence (Eysenck, 1967; Duffy, 1966) to assume that physiological arousal induced by competition might be related to personality traits, especially extroversion and neuroticism. Taking into account Eyscenk’s (1967) argument, which relates neuroticism to the arousability of a system for emotions centered on the limbic system, it can be postulated that neuroticism should be related to greater physiological arousal before an important competition. Based on Ode and Robinson’s (2007) statement indicating that agreeableness might be a personality trait that may have the potential to regulate neuroticism related distress, it can be suggested that agreeableness might be another personality trait that can be associated with lower pre-competition physiological arousal. There is also strong evidence to assume a negative association between pre-competition physiological arousal and conscientiousness. In a previous study, conscientiousness has been found to be associated with reduced daily cortisol concentration and more frequent positive affect (Nater et al., 2010). Further, Bartley and Roesch (2011) showed that high conscientiousness individuals tended to use problem focused coping which can lead to a positive effect.

The argument made by Matthews (1985), which suggests that specifying the relationship between personality traits and arousal may help to predict the effect of personality on performance, can constitute another logical basis for this study. The examination of the relationship between cognitive anxiety, physiological arousal, and personality may lead to better predictions of athletic performance.

On the basis of the reasons mentioned earlier, this study aims to examine the relationship between cognitive anxiety, personality traits, and physiological arousal induced by competition. Considering the statement by Barrett and Armony (2006), which suggests that with anxiety, the absence of a real threat or the inappropriateness of fight-or-flight behavior renders physiological changes, we postulated that physiological arousal evoked by competition should be more closely related to personality traits than anxiety. Thus, pre-competition physiological arousal should be negatively related to agreeableness and conscientiousness.

## Material and Methods

### Participants

A total of 30 taekwondo athletes (20 male and 10 female), ranging in age from 18 to 20 years (mean age = 18.6 years; ± 1.8), who participated in national team selection competitions, volunteered to participate in the study. Participants gave written, informed consent to participate in the study. All data in this study were collected in accordance with the ethical standards of the Helsinki Declaration.

### Cognitive Anxiety

To measure cognitive anxiety, the state version of the Spielberger State-Trait Anxiety Inventory (STAI) (Spielberger et al., 1970) was used. The scale features 20 items that require a response using a four-point Likert-type scale.

### Personality

To assess personality characteristics, the Five Factor Personality Inventory (FFPI) developed by Somer et al. (2002) was used. The FFPI is a 220-item personality inventory designed to assess five main personality traits: neuroticism, extraversion, openness to experience, agreeableness, and conscientiousness, as well as 17 subdimensions. Item responses were made using a five-point format. The inventory’s manual provides evidence for the acceptable reliability and validity of the measurement device.

### Electrodermal Activity (EDA) Recording

To evaluate physiological arousal, EDA was recorded in an isolated room with an ambient temperature of 23º C. The Biopac (MP150) data-acquisition system was used to record EDA signals. Measurements were made using Ag/AgCl electrodes that were placed on the distal pads of the second and third fingers of the non-dominant hand. Electrodes were filled with a 0.05% NaCl electrode paste. The data were sampled at 200 Hz and stored on computer disks for subsequent analysis. Participants’ EDA was recorded for 300 seconds. The first and last 30-second periods were not included in later analyses. Signals were expressed as micromho (μmho). Delta values were taken into account to determine athletes’ arousal level. Delta EDA value was computed by taking the difference between consecutive samples and filtering out the negative values. When summed over a period of time, the delta EDA served as a metric for the athletes’ total arousal for the time period (Shye et al., 2008). The range of the delta EDA depended upon the amplitude of the selected time period.

### Procedure

One day prior to the national team selection games, athletes were invited to participate in the study and 37 of them agreed to participate in the study. However, seven athletes were excluded from the study because of technical problems such as power being cut during the recording and a high level of common mode signals (noise). The study was executed in two phases. In the first phase, participants completed the FFPI. Their resting EDA was then recorded. All components of the first phase were executed before the weight and drawing lots segments of the selection process. The second phase of the study was conducted on the first day of the selection games. In this phase, participants’ EDA was recorded approximately one hour before their first match. After recording EDA, the participants were allowed to leave for their warm-up sessions. They also completed the state scale of the STAI approximately 10 minutes before the competition.

### Statistical analysis

In order to analyze the descriptive statistics of the obtained data set, the Pearson moment correlation coefficient and regression analyses with stepwise method were used. SPSS 11.0 was used in statistical analyses.

## Results

As can be shown in Table 1, the Pearson correlation coefficient was calculated to examine the relationship between personality traits, cognitive anxiety and EDA delta scores. The results showed a negative and significant correlation between the personality trait of agreeableness and the EDA delta scores measured one day before the competition (r = −0.43, p = 0.017). EDA delta scores measured one day before the competition were also significantly and positively correlated to neuroticism (r = 0.45, p = 0.012). The Mean EDA delta score for the day prior to competition was 0.18 μmho. In order to test whether personality traits could significantly predict variations in EDA delta scores, a stepwise regression analysis was conducted. The final model containing only neuroticism significantly explained the variations in EDA scores measured one day before the competition (*R^2^* = 0.21; *F*_(1, 28)_ = 7.25, *p* = 0.012).

Personality traits were also associated with EDA delta scores measured one hour before the competition. The results showed that there were negative and significant correlations between agreeableness-conscientiousness and EDA delta scores measured one hour before the competition (r = −0.50, p = 0.005; r = −.50, p = 0.006). No significant relationship was observed between the cognitive anxiety state and EDA delta scores measured one hour before the competition. The Mean EDA delta score for the measurement taken one hour prior to competition was 0.29 μmho.

To test whether personality traits could significantly predict variations in EDA delta scores measured one hour before competition, a stepwise regression analysis was conducted. It was found that the final model containing only agreeableness significantly explained variations in EDA delta scores (*R^2^* = 0.25; *F*_(1, 28)_ = 9.01, *p* = 0.012).

## Discussion

The main aim of this study was to examine the relationship between personality traits, cognitive anxiety, and physiological arousal induced by competition. The results indicate that physiological arousal measured one day before the competition - when the athletes face a number of stressors such as drawing lots and weighing to determine a rival, long distance travel or possible sleep deprivation might be related to agreeableness and neuroticism. As expected, neuroticism was positively related to EDA delta scores, which means that those athletes who have higher levels of neuroticism may be more prone to physiological arousal than their low neuroticism counterparts. This result was not surprising, considering that the results of Norris et al. (2007) showed that autonomic nervous system indicators such as electrodermal and electrocardiac responses are associated with neuroticism. Results of the present study might also be considered as evidence supporting Eysenck’s (1967) personality theory, which relates neuroticism to the reactivity of the limbic system and low stress tolerance.

Interestingly, neuroticism was not related to physiological arousal measured one hour before the competition. This lack of association can possibly be explained by the disappearance of some of the stressors. For example, several participants were then certain of what the task was in front of them, which may have produced a lower anxiety level.

This lack of association between neuroticism and physiological arousal in a low anxiety state can be shown as evidence to support the notion that in the case of high anxiety, neuroticism might be related to a higher level of physiological arousal.

In contrast to other fields of psychology, sport psychology has rarely examined the effect of personality traits in understanding anxiety and other related physiological responses. However, the association between physiological arousal measured one day before competition and neuroticism reveals some implications for sport psychology. First, neuroticism might be a useful personality trait for understanding athletes’ predisposition to anxiety. In addition, neuroticism might be used to predict athletes’ physiological responses to competition. Thus, this information may be useful in the regulation of athletes’ arousal levels.

Another interesting result observed in this study was the association between physiological arousal induced by competition and agreeableness. Agreeableness was moderately and negatively related to EDA delta scores measured both one day and one hour before the competition, which means that athletes who scored high on this trait may have tended to be more stable in terms of peripheral nervous system activity. Agreeableness has been largely examined in terms of interpersonal relationships (Moskowitz, 1994; Jensen-Campbell and Malcolm, 2007) and, to our knowledge, no previous study has examined the possible link between psychophysiological arousal and agreeableness in sports. However, the results of the present study, along with those of previous studies from other fields of psychology, indicate that agreeableness may contribute to the predictive ability of models to clarify the relationship between competitive anxiety, related psychophysiological states, and athletic performance. For example, Ode and Robinson (2007; 2009) clarified the role of agreeableness in regulating negative emotions, which means that individuals who scored high on this trait may be prone to having lower levels of anxiety in response to an important competition.

A study examining the psychophysiological correlates of agreeableness provides partial evidence for the moderate negative association between EDA delta scores and agreeableness observed in the current study. In this respect, Stough et al. (2001) found agreeableness to be associated with EEG beta-1 activity in the left central temporal region. In a previous study (Shagass, 1955), it was shown that beta-1 activity is related to anxiety and that although EDA is an autonomous response of the peripheral nervous system, it can be influenced indirectly by central nervous system activity. On the basis of the results of the current study, along with those of previous studies demonstrating negative association among agreeableness, anxiety, and physiological arousal, we concluded that agreeable athletes may possibly have a higher level of arousal threshold than non-agreeable athletes do. Moreover, agreeableness may lead athletes to be more stable in terms of peripheral nervous system activity in response to an important competition.

In the current study, conscientiousness is found to be another personality trait that might be related to more stable peripheral nervous system activity in response to an important competition. There might be several reasons for more stable EDA activity in athletes with higher levels of conscientiousness, and one of the most important reasons for a lower level of physiological arousal might be related to a facet of conscientiousness. Considering the statements of Chamorro-Premuzic and Furnham (2004) and Furnham et al. (2002) describing conscientious individuals’ characteristics, it might be logical to expect an association between conscientiousness and a lower level of physiological arousal. Athletes who are persistent, self-disciplined, achievement-oriented, organized, attentive and focused on obtaining good results may have a relatively higher possibility of success in competition and, as a result, might be less psychophysiologically aroused. However, these results regarding the relationship between conscientiousness and psychophysiological arousal should be interpreted with caution owing to the research findings demonstrating a link between conscientiousness and beta-1 activity (Stough et al., 2001) that is related to anxiety (Shagass, 1955). In addition, there is evidence to assume a positive association between conscientiousness and anxiety in certain circumstances. For example, Cianci et al. (2010) found in their experiment that high conscientiousness may facilitate performance only in individuals with a learning goal. They also showed that individuals with high conscientiousness experienced greater tension than individuals with low conscientiousness in both performance and learning tasks. In this respect, the relationship between conscientiousness, anxiety, and related physiological arousal might be mediated by an athletes’ goal orientation. Therefore, in future studies, athletes’ goal orientation should be taken into account to further understand personality, anxiety, and psychophysiological arousal association.

The final finding is the lack of association between cognitive anxiety and psychophysiological arousal. As expected, there was no relationship between cognitive anxiety and psychophysiological arousal. Several explanations might be offered to explain the lack of this relationship between cognitive anxiety and psychophysiological arousal. First, in anxiety, the absence of a real threat or the inappropriateness of fight-or-flight behavior renders physiological changes (Barrett and Armony, 2006). Second, considering the argument of Beattie and Davies (2010), it can be suggested that the relationship between cognitive anxiety and psychophysiological arousal might be mediated by self-confidence.

## Conclusions

The results of the current study show that taking personality traits into account, especially those within the Big Five personality model, may lead to a better understanding of athletes’ psychophysiological arousal level. This information can contribute to the predictive ability of the theoretical models aiming to understand arousal-performance relationships. In addition, the current results indicate that practitioners dealing with the regulation of athletes’ arousal level should consider athletes’ personality characteristics.

However, this study may have some limitations. First, in the current study it was assumed that EDA measured one day prior to the event could represent the physiological arousal induced by competition. However, other factors such as travel, climate and sleep deprivation may possibly effect the physiological arousal measured one day prior to the event. Second, the data obtained in this study does not allow for a distinction between successful and less successful athletes in terms of psychophysiological arousal. In future studies, the authors suggest testing the relationship between anxiety, physiological arousal, and athletic performance in well-controlled experimental studies.

## Figures and Tables

**Table 1:** Correlations between personality traits and EDA delta scores

	One day prior EDA	One hour prior EDA
1-Extroversion	0.17	0.01
2- Agreeableness	−0.43^**^	−0.50^**^
3- Conscientiousness	−0.14	−0.50^**^
4 -Emotional Stab.	0.45^*^	0.13
5- Openness	0.07	−0.20
6 -State anx.	0.04	0.04
